# The clinicopathological features, treatment outcomes and follow-up results of 47 ectopic thyroid gland cases: a single-center retrospective study

**DOI:** 10.3389/fendo.2023.1278734

**Published:** 2023-11-24

**Authors:** Ming Gao, Qi He, Liwen Li, Feihong Ji, Yalei Ding, Qixuan Sun, Xinguang Qiu

**Affiliations:** The Department of Thyroid Surgery, the First Affiliated Hospital of Zhengzhou University, Zhengzhou, Henan, China

**Keywords:** ectopic thyroid gland, accessory thyroid, aberrant thyroid, follow-up, retrospective study

## Abstract

**Background:**

Ectopic thyroid gland (ETG) is an uncommon clinical condition, presenting various challenges and limitations in its regulate diagnosis and treatment currently. This study aims to enhance our understanding of ETG and improve the strategies for its diagnosis and treatment.

**Methods:**

The retrospective single-center study was conducted, encompassing clinical data from ETG patients screened at our institution between 2013 and 2022. Patients were categorized based on the location of the disease, and follow-ups were performed on each.

**Results:**

This study included a total of 47 patients who were confirmed to hav confirmed to have ETG. Among them, we found 29 cases of accessory thyroid and 18 cases of aberrant thyroid. Furthermore, 42 cases exhibited the single ETG, while 5 cases displayed the double ETG. The distribution of the ETG was as follows: 20 were lingual, 10 were submandibular, 10 were lateral cervical, 4 were thoracic mediastinal, 1 was esophageal, and 7 were ovarian. Of these cases, 22 patients underwent surgery, 18 received thyroid hormone replacement therapy, and 7 were placed under observation. All patients were followed up for 59.4 (12-117) months. No significant abnormalities were detected at the conclusion of the follow-up period.

**Conclusion:**

ETG is frequently observed in the head and neck, particularly in lingual. Accessory thyroid glands are commonly reported, with most cases being single ETG. Notably, these glands usually do not manifest specific clinical symptoms. Therefore, the appropriate and comprehensive examinations during the initial diagnosis are crucial to avoid misdiagnosis. Treatment should be individualized, and long-term follow-up is essential for managing ETG effectively.

## Introduction

Ectopic thyroid gland (ETG) is a rare congenital disorder in which thyroid tissue is located outside the typical position of the thyroid gland in the neck (anterior to the second to fourth tracheal rings) (1). The incidence of ETG is approximately 1/100,000 ~ 1/300,000. It can occur in people of all age groups but is more common in children and middle-aged patients, especially in women. Typically, ETG lacks specific clinical symptoms and can locate at any position from the midline of the neck to the midline of the sternal notch. Among the various types of ETG, lingual thyroid is the most frequently encountered, accounting for approximately 90% of cases (2–4).

Embryologically, the thyroid primordium originates from the primitive pharynx and neural crest and generally begins its development during the 3rd to 4th week of the gestation. It migrates during the 5th to 7th week of embryonic development. When the migration is abnormal, and the thyroid primordium fails to reach its usual anatomical position in front of the trachea, an ETG is formed (5). However, ETGs in some specific locations such as adrenal glands and liver remain unexplained embryologically yet. While there is no well-established molecular mechanism for ETG development, some key transcription factors associated with thyroid maturation and differentiation genes, such as TITF1, Tg, PAX8, and FOXE1, may play a significant role. Mutations in these genes can lead to abnormalities in thyroid morphogenesis and migration process (3, 6).

Most ETGs typically show a single ectopic thyroid tissue, although double and triple ectopic thyroid are rarer (7). ETG can be classified into two types based on development. Those lacking thyroid tissue in the normal thyroid position in the neck are referred to as aberrant thyroid gland (ABTG), accounting for approximately 25% of case (8, 9). In contrast, ETG in which thyroid tissue coexists with the normal thyroid in the neck is termed accessory thyroid gland (ACTG) and is more commonly observed (8, 9). ETG has a limited ability to absorb iodine and frequently exhibit insufficient thyroid function compared to normal thyroid tissue. Consequently, it is often accompanied by hypothyroidism, while cases of hyperthyroidism are occasionally reported. Additionally, ETG has a low incidence of cancer development, without any specific features of the various pathologic types (10). In clinical, ETG is often subject to misdiagnosis and inappropriate treatment. So it is necessary to enhance the understanding of it and to develop an appropriately individualized treatment plan. Our study represents the world’s largest single-center sample size of comprehensive ETG diagnosis and treatment processes to date. We retrospectively analyzed their clinical and pathological features, as well as the diagnostic and treatment processes, observed the long-term prognosis of various treatment outcomes. This study aims to contribute valuable experience to the diagnosis and treatment of ETG.

## Materials and methods

### Subjects

This study collected data of patients with ETG admitted to the First Affiliated Hospital of Zhengzhou University between 5/2013 to 2/2022. Including gender, age, clinical symptoms, ETG location, thyroid function, treatment methods, and pathological features, etc.

**Figure 1:**
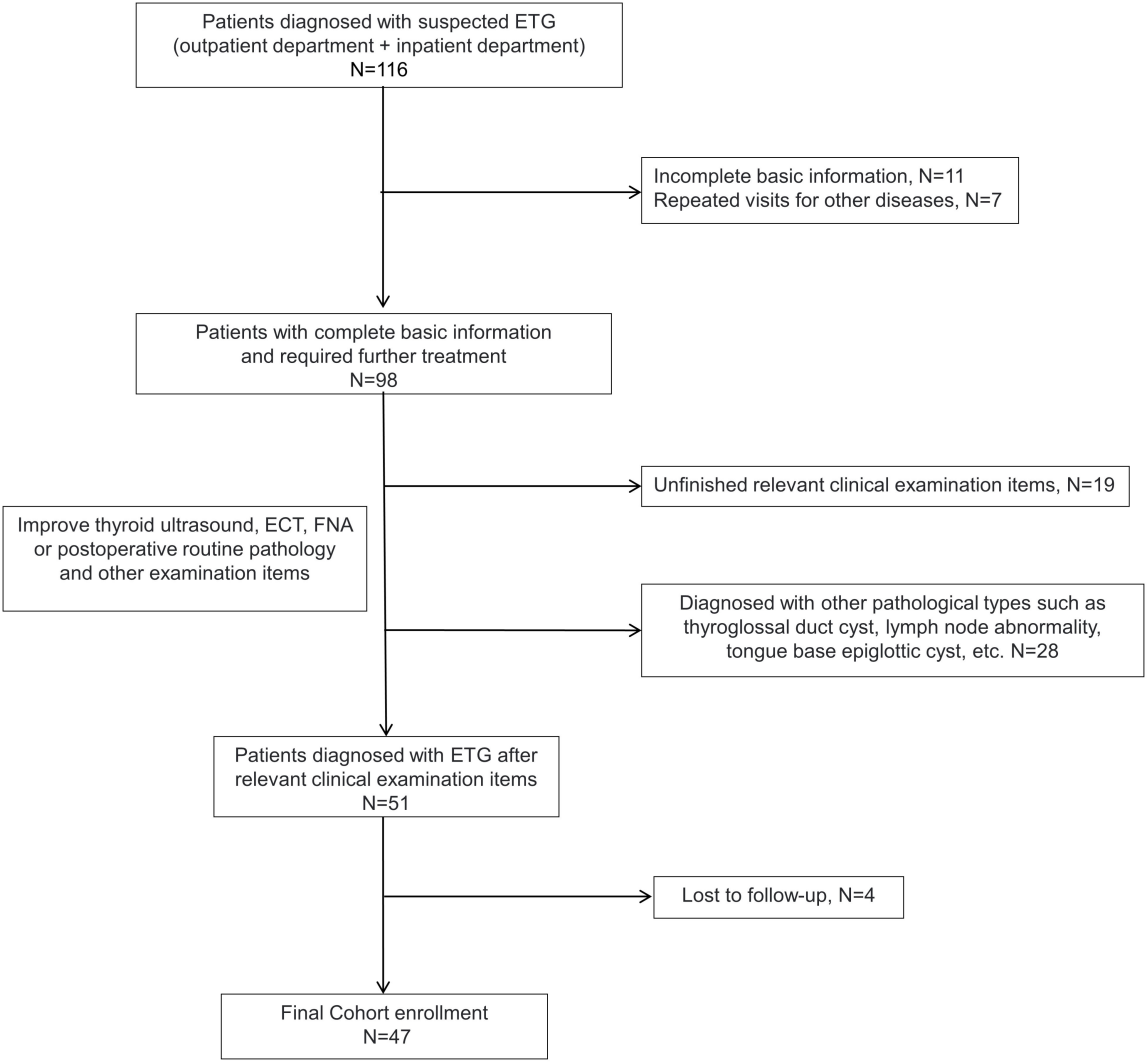
Flow chart of clinical data inclusion in 47 cases of ETG.

Inclusion criteria for ETG diagnosed at least one of the following: (1) positive thyroid static imaging (^99^Tc^m^ or I^131^), (2) ultrasound-guided fine-needle aspiration cytology (FNAC) confirmed, (3) postoperative routine pathology confirmed. The exclusion criteria were (1) ultrasound suggestive of suspicious ETG but negative thyroid static imaging, (2) other pathologic types such as thyroglossal cysts, (3) Incomplete clinical data or patients lost during follow-up (**Figure 1**).

### Treatment and follow-up methods

Besides routine examinations, patients underwent a comprehensive diagnostic workup, which included thyroid ultrasound, thyroid static imaging, CT, MRI, and laryngoscopy. The choices of observation, thyroid drug therapy and surgery were made individually based on patients’ unique clinical symptoms and personal preferences.

Regular follow-up reviews were performed during the observation period. Thyroid drug therapy was thyroxine tablet replacement therapy. The surgical approach was decided by the nature and location of the ETG. Follow-up was via telephone and visit records, with the initial date of diagnosis and the final follow-up time recorded as the cut-off date. Follow-up assessments encompassed ultrasound examinations of the thyroid and ectopic sites, CT and thyroid function, etc.

## Results

**Figure 2:**
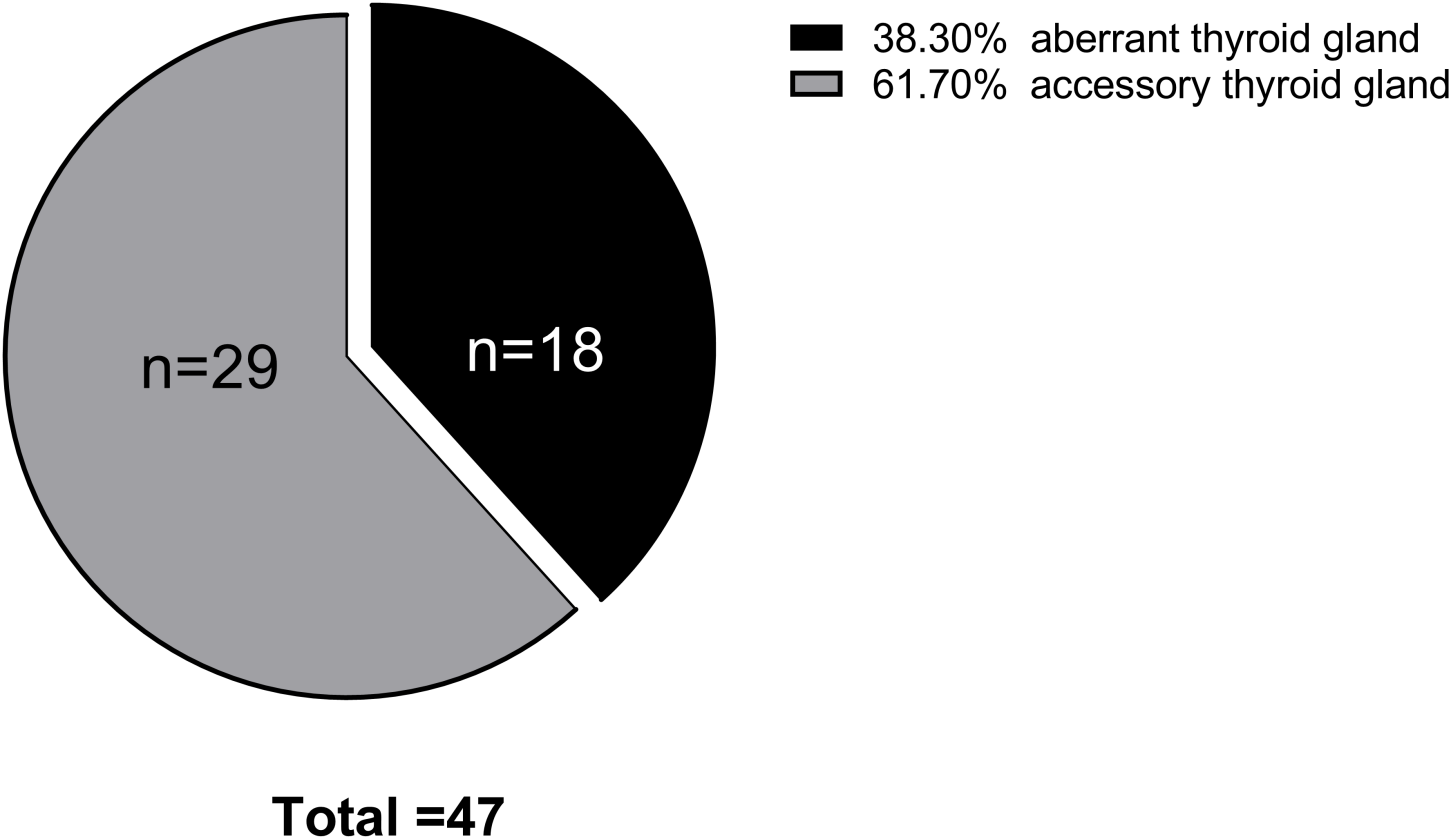
Pie chart of ETG type distribution in 47 cases.

**Figure 3:**
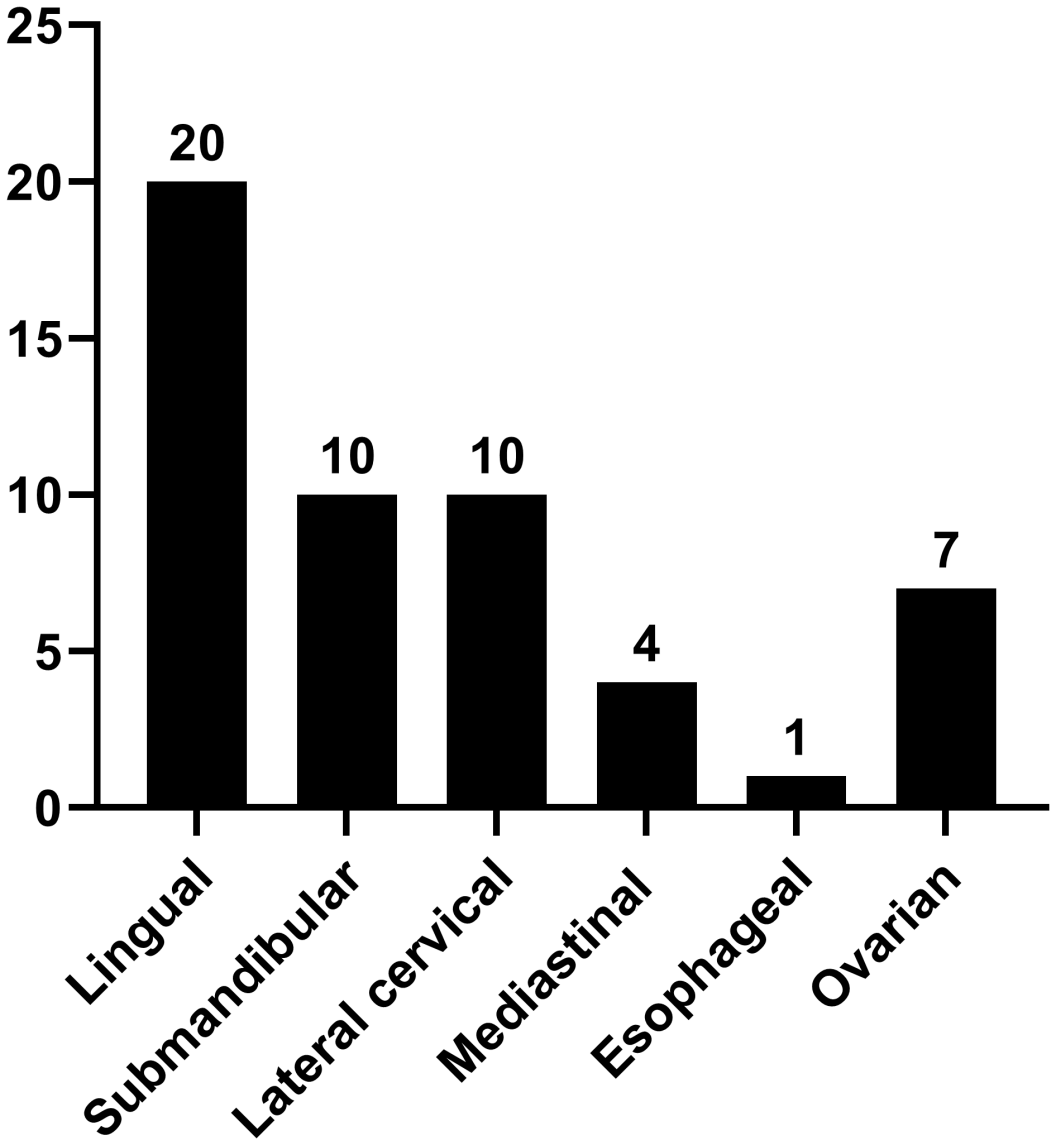
Frequency histogram of the distribution of 52 ETG gland locations.

The study included 47 patients diagnosed with ETG totaling 52 ectopic thyroid glands. Among these, 31 ectopic glands (59.6%) were palpable, and 26 patients were symptomatic. Notably, the majority of ETG patients were female, accounting for 37 individuals (78.7%). The mean age was 36.0 (0.4-65) years old, with 10 patients being underage. The average maximum diameter of the ectopic glands was 32.9 (3-116) mm, and the average volume was 41685.3 (81-592875) mm^3^. Among the cases, 29 (61.7%) were ACTG, while the remaining 18 cases were ABTG (**Figure 2**). Of the total cases, 42 (89.4%) featured a single ETG, 5 cases (10.6%) presented with double ETGs. The distributions of the ETGs were as follows: 20 were lingual, 10 were submandibular, 10 were lateral cervical, 4 were thoracic mediastinal, 1 was esophageal, and 7 were ovarian (**Figure 3**). In cases of single ETG, 19 patients underwent surgery, 16 were given Thyroid Hormone Replacement Therapy (THRT), and 7 were placed under observation. For the other 5 cases of double ETG, 3 cases were treated with surgery, followed by THRT. And the other 2 cases were managed with THRT. All patients were followed up for an average of 59.4 (12-117) months. At the end of the follow-up period, no significant abnormalities were observed.

### Single ETG

42 cases of single ETG were reported, comprising 89.4% of the total cases. Their clinical characteristics are detailed in **Table 1**, examination results are presented in **Table 2**, and treatment and follow-up results can be found in **Table 3**. Due to the specific clinical manifestations and diagnostic modalities of ETG in various anatomical locations, our study categorized and described them comprehensively.

**Table 1:** The clinical data and symptoms of 42 patients with single ETG.

Variable	ETG N=42						
	Lingual and Sublingual N=17	Submandibular N=7	Lateral cervical N=6	Mediastinal N=4	Esophageal N=1	Ovarian N=7	Total N=42
Gender							
**Male**	3	2	1	1	1	0	8
**Female**	14	5	5	3	0	7	34
Age (years)							
**<18**	2	4	1	0	0	0	7
**≥18**	15	3	5	4	1	7	35
**average age (years)**	30.5	20.0	48.3	55.5	64.0	49.7	36.7
Maximum diameter (mm)							
**<20**	6	2	1	0	1	0	10
**≥20**	11	5	5	4	0	7	32
Type of ETG							
**Aberrant thyroid gland**	12	4	1	0	0	0	17
**Accessory thyroid glands**	5	3	5	4	1	7	25
Thyroid surgery history							
**Yes**	1	1	1	0	0	0	3
**no**	16	6	5	4	1	7	39
Clinical symptoms							
**Cough and sputum**	2	0	0	1	0	0	3
**Foreign body sensation**	7	1	0	0	1	0	9
**Dysphagia**	5	0	0	0	0	0	5
**Pressing pain**	1	1	0	0	0	1	3
**Voice hoarse**	2	0	0	0	0	0	2
**Palpable mass**	5	7	3	0	0	2	17
**Asymptomatic**	6	0	3	3	0	4	16
Way to discover the suspected ETG							
**Physical examination**	6	0	3	3	0	4	16
**Clinical symptoms**	11	7	3	1	1	3	26

**Table 2:** Examination test results of 42 patients with single ETG.

Variable	ETG N=42						
	Lingual and Sublingual N=17	Submandibular N=7	Lateral cervical N=6	Mediastinal N=4	Esophageal N=1	Ovarian N=7	Total N=42
Thyroid function							
**Undone**	0	0	0	0	0	2	2
**Done**	17	7	6	4	1	5	40
** Normal**	3	4	3	4	1	4	19
** Hypothyroidism**	13	2	1	0	0	1	17
** Hyperthyroidism**	1	0	1	0	0	0	2
** TSH Suppressed state**	0	1	1	0	0	0	2
Sex hormones							
**Undone**	11	7	6	4	1	0	29
**Done**	6	0	0	0	0	7	13
** Normal**	2	0	0	0	0	7	9
** Hyperprolactinemia**	4	0	0	0	0	0	4
Imaging examination							
**Cervical ultrasound**	16	7	6	2	1	5	37
**Abdominal ultrasound**	7	0	0	1	0	7	15
**Tc^99m^ imaging**	14	5	2	0	0	0	24
**CT**	8	5	4	4	1	3	24
**MRI**	8	0	0	0	0	1	9
**gastroscope**	1	0	0	0	1	0	2
**laryngoscope**	6	0	0	0	0	0	6
**FNA**	0	0	2	3	0	0	5

**Table 3:** Treatment follow-up outcomes of 42 patients with single ETG.

Variable	ETG N=42						
	Lingual and Sublingual N=17	Submandibular N=7	Lateral cervical N=6	Mediastinal N=4	Esophageal N=1	Ovarian N=7	Total N=42
Treatment Programs							
**Surgical**	3	2	5	1	1	7	19
**Observation**	2	2	0	3	0	0	7
**Thyroxine replacement/TSH Suppression therapy**	12	3	1	0	0	0	16
Diagnostic method							
**Imaging**	14	5	1	0	0	0	20
**Pathology**	3	2	5	4	1	7	22
Pathological results							
**Thyroid tissue**	15	6	1	4	1	0	27
**Nodular goiter**	1	1	5	0	0	7	14
**Papillary thyroid carcinoma**	1	0	0	0	0	0	1
**Follow-up time (months)**	52.7	93.0	29.0	39.5	103.0	63.3	57.7
Follow-up outcome							
**Normal thyroid function**	16	6	4	4	1	7	38
** Observe persistently**	3	4	0	4	1	7	19
** Thyroid hormone replacement therapy**	13	2	4	0	0	0	19
**TSH Suppression status**	1	1	2	0	0	0	4

### Lingual ETG

**Figure 4:**
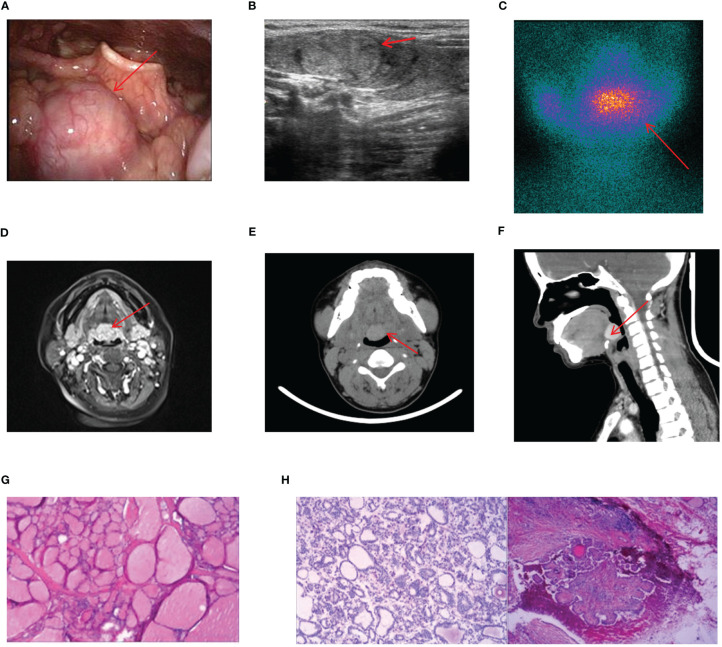
Imaging manifestations and pathological findings of lingual ETG. **(A)**: The laryngoscope showed that there is a light red or red hard mass at the base of the tongue with smooth surface and clear boundaries. **(B)**: The thyroid ultrasound showed a rounded solid, cystic, or cystic-solid echo at root of the tongue. **(C)**: Static imaging of thyroid gland suggested that a circular abnormal aggregation in the distribution of radioactivity at the root of the tongue. **(F)**: Both MRI and CT showed a circular mass at the root of the tongue. **(G)**: Postoperative pathologic result of a lingual ETG patient suggested the goiter, immunohistochemical results: TTF-1 (+), TG (+), Pax-8 (+), SOX-10 (-), Dog - 1 (-). **(H)**: Postoperative pathologic result of a lingual ETG patient suggested the PTC. **(D, G)** were of the same patient, **(B, H)** were of the other same patient, and **(E, F)** were of another same patient.

17 cases of lingual ETG were reported, comprising 14 females and 3 males, with ages ranging from 5 months to 51 years old and the mean age of 30.5 years old. Among these cases, 12 were ABTG and 5 cases were ACTG. While 9 cases were found on their own or by daily physical examination incidentally, the remaining 8 cases were diagnosed with obvious clinical symptoms. The most common complaint was a foreign body sensation in the larynx, and other complaints included dysphagia, hoarseness, and coughing up sputum. Only one patient complained of localized pressure and pain. Among the 17 cases where thyroid function was assessed, hypothyroidism was observed in 13 cases, one case showed subclinical hyperthyroidism, and the remaining 3 cases had normal thyroid function. Sex hormone tests were performed in 6 cases, with 4 of them suggesting hyperprolactinemia. Laryngoscopy, ultrasound, CT, and other imaging findings were shown specifically (**Figures 4A–F**). For 14 cases were confirmed by ^99^Tc^m^ thyroid static imaging. Of these, 12 were given THRT for hypothyroidism and 2 were observed due to their normal function. The remaining 3 cases underwent tongue base neoplasmectomy under support laryngoscope. They were diagnosed by postoperative pathology as 1 case of papillary thyroid carcinoma (PTC), 1 case of thyroid tissue, and 1 case of nodular thyroid goiter (**Figures 4G, H**). At the end of follow-up (mean follow-up, 52.7 months), one patient with PTC remained in a TSH-suppressed state, while the remaining 16 cases exhibited normal thyroid function with no apparent imaging abnormalities.

### Submandibular ETG

**Figure 5:**
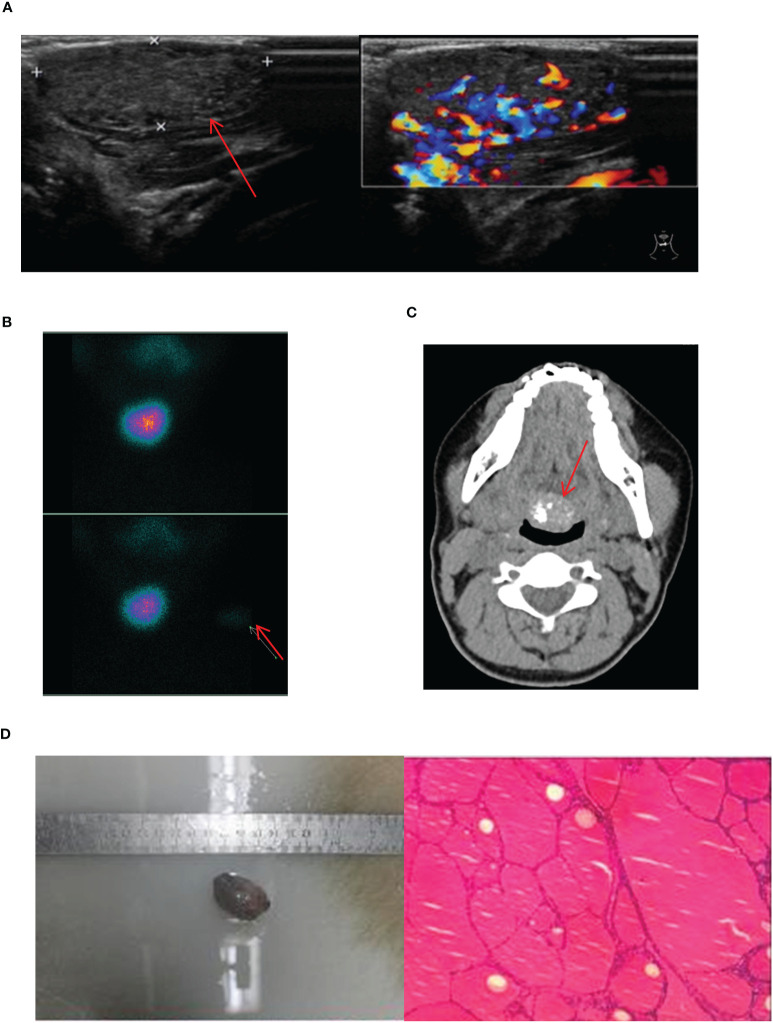
Imaging manifestations and pathological findings of submandibular ETG. **(A)**: The thyroid ultrasound showed a hypoechoic glandular echo with clear boundaries, regular shape. The Color Doppler flow imaging showed abundant blood flow signals in the ETG. **(B)**: Static thyroid imaging showed abnormal circular radioactive distribution under the mandible. **(C)**: CT showed a soft tissue density shadow of nodules with clear boundaries in front of the trachea under the mandible, which was obviously enhanced after enhancement. **(D)**: Postoperative pathology indicated the thyroid tissue. **(C, D)** were of the same patient.

In this study, we uniformly categorized the cases found in the midline of the neck between the thyroid cartilage and the hyoid bone as the submandibular ETG. There were 7 cases of submandibular ETG, consisting of 5 females and 2 males with an average age of 20 years old. For these cases, 4 were juvenile patients with ABTG, and 3 were adult patients with ACTG. Interestingly, all patients presented at the hospital with a self-detected submandibular mass. And one of them was reported a significant foreign body sensation, another experienced localized compression pain, and one had a suspicious anterior cervical mass detected 10 years prior to presentation. The thyroid function results revealed hypothyroidism in 2 cases, and the remaining cases exhibited normal thyroid function. 5 cases were confirmed through ^99^Tc^m^ imaging, comprising 4 cases of thyroid tissue and 1 case of nodular thyroid goiter, respectively (**Figure 5**). The remaining 2 cases underwent neck resection, and postoperative pathology confirmed the diagnosis of thyroid tissue (**Figure 5D**). Throughout a mean follow-up time of 93.0 months, no recurrence or progression were observed.

### Lateral cervical ETG

**Figure 6:**
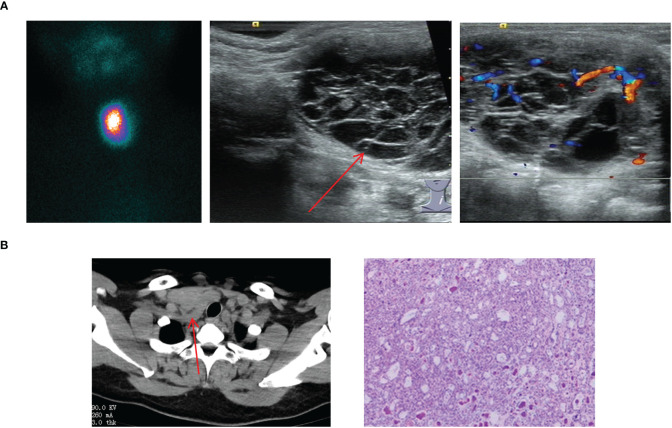
Imaging manifestations and pathological findings of lateral cervical ETG. **(A)**: A 7-year-old girl with a cystic-solid mass in the lymph node of zone IV. The thyroid ultrasound showed a cystic-solid echo with clear boundaries, which had visible blood flow signals; the static thyroid imaging showed abnormal radioactive distribution in the neck. **(B)**: A 51-year-old woman, the postoperative pathology of her right neck mass presented with the adenomatoid nodular goiter. The CT showed a mass with soft tissue density shadow behind the right neck clavicle that had the irregular shape and unclear boundary.

In this study, ETG found below the level of the thyroid cartilage and above the sternal notch were classified as the lateral cervical ETG. We documented a total of 6 cases of lateral cervical ETG, comprising 5 females and 1 male, with age ranging 7-65 years old. Only 1 case involved a pediatric patient with ABTG, while the remaining 5 adult patients were ACTG. The common ultrasound characteristics in these cases were a cystic solid or solid mass with clear edges and visible blood flow signals within. Static thyroid imaging was performed in two of these cases, demonstrating a distinct soft tissue shadow of technetium reflux in the neck at the location of palpation of the mass (**Figure 6**). Thyroid function tests indicated hypothyroidism in one case and subclinical hyperthyroidism in another. Among the 6 cases of lateral cervical ETG, 5 underwent cervical mass resection + ipsilateral thyroidectomy + central lymph node dissection. All of them were diagnosed as nodular thyroid goiter by postoperative pathology, and one case also combined with isthmus PTC. With a mean follow-up of 29.0 months, two patients were in a state of TSH suppression while the remaining patients were administered THRT.

### Mediastinal ETG

**Figure 7:**
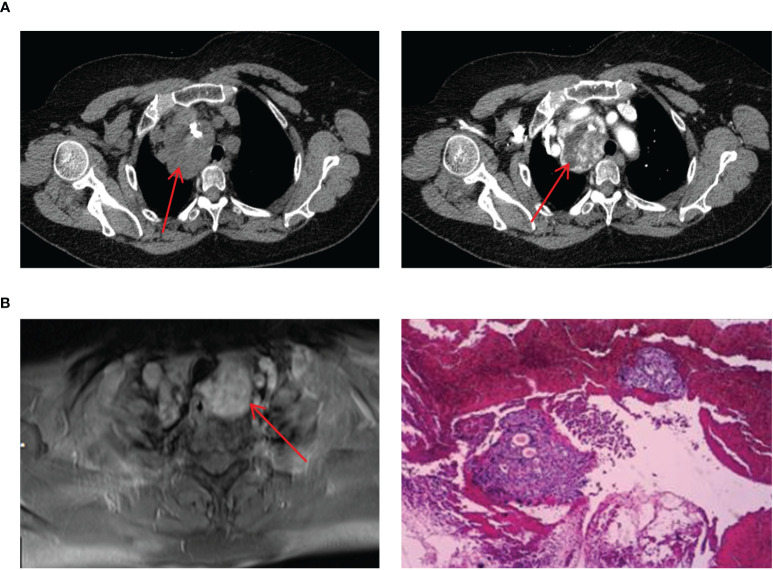
Imaging manifestations and pathological findings of mediastinal ETG. **(A)**: CT showed a soft tissue mass with clear boundaries, the esophagus and trachea were compressed and displaced, and enhancement could be seen. **(B)**: MRI showed a mass with the high-density image and pathology of FNAC indicated ETG, immunohistochemical results: AE1/AE3 (+), TTF-1 (+), CK7 (+), Pax-8(+), TG (+), PTH (-), Galectin-3(-).

The four cases of mediastinal ETG in our study, consisting of 3 females and 1 male, with ages ranging from 47 to 61 years old. 2 cases presented with ectopic thyroid growths extending into the upper mediastinum, one on each side. The remaining two cases featured independent growths in the upper mediastinum, one posterior to the trachea and the other in the anterior mediastinum at the site of the thymus (**Figure 7**). All 4 cases were classified as ACTG, and they had normal thyroid function. The diagnosis of thyroid tissue was confirmed by FNAC in 3 cases, and the remaining 1 case underwent thoracoscopic resection of the mass. Postoperative pathology confirmed the combination of thyroid tissue with thymic lipoma. Throughout a mean follow-up time of 39.5 months, none of the 4 patients received THRT, and all maintained normal thyroid functions. Imaging studies showed no significant abnormalities.

### Esophageal ETG

**Figure 8:**
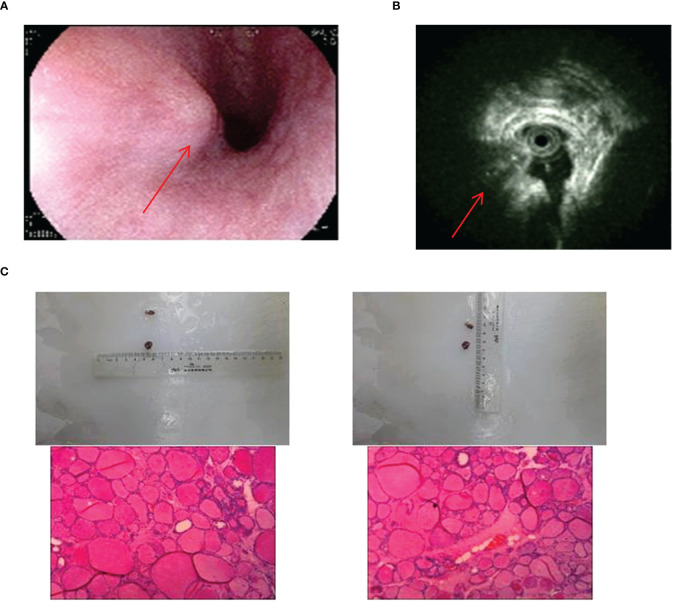
Imaging manifestations and pathological findings of esophageal ETG. **(A)**: Gastroscopy showed a semi-spherical, smooth-surfaced mucosal bulge on the anterior wall of esophagus which was 18cm far from the incisor. **(B)**: The endoscopic ultrasonographic finding showed that the level of the esophageal wall was normal, and an ectopic tissue echo was found outside the esophageal wall. **(C)**: The postoperative pathology showed thyroid tissue.

There was only one case of esophageal ETG in this study, which was classified as ACTG. The patient was a 64-year-old male who presented with a persistent dysphagia lasting for a year, and had normal thyroid function. Upon examination via CT, a slight thickening of the wall of the upper esophagus was observed, which exhibited a clear demarcation from surrounding structures (**Figure 8**). The patient underwent resection of the cervical esophageal mass, and was not given THRT after postoperative pathology confirmed. At the conclusion of a 103-month follow-up period, both thyroid function and imaging studies revealed no significant abnormalities.

### Ovarian ETG

**Figure 9:**
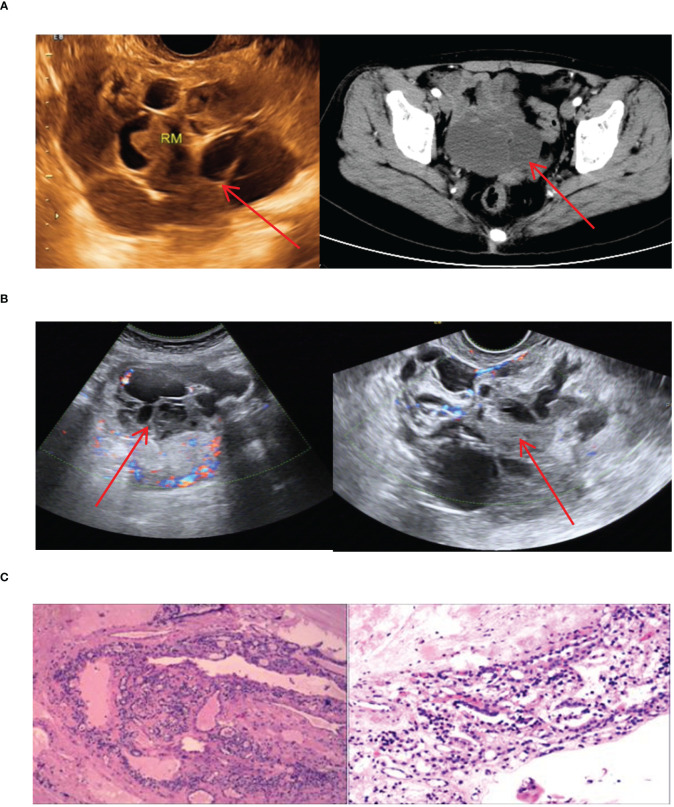
Imaging manifestations and pathological findings of ovarian ETG **(A, B)**: Both ultrasound and CT images showed a cystic or cystic-solid mass in the adnexal area, which had poor confluency and multiple cystic cavities of different sizes and irregular shapes. **(C)**: The postoperative pathology showed the nodular goiter in the ovary. Both ultrasound and CT showed a cystic or solid cystic mass in the adnexal area with poor fusion and multiple irregularly shaped sacs of different sizes.

Our study totally had 7 cases of ovarian ETG, with 4 cases located on the right side and 3 on the left side. These cases ranged in age from 44 to 63 years old, and all were classified as ACTG. Among these cases, 2 patients presented with palpable abdominal masses, 1 reported for abdominal pain, and the remaining 4 cases were incidentally found in routine medical checkups with suspicious ovarian masses. It’s worth noting that none of the ovarian ETG cases were suspected preoperatively. As a result, static thyroid imaging was not performed (**Figure 9**). Thyroid function tests were performed in 5 cases, 1 case had hypothyroidism and the remaining 4 cases were normal. Additionally, no significant abnormalities were observed in any of the sex hormones tests. All patients underwent surgical resection, and postoperative pathology confirmed the presence of nodular thyroid goiter. Furthermore, the postoperative thyroid function of the 7 patients were normal. Until the end of follow-up period (mean follow-up time 63.3 months), thyroid function remained normal in all 7 cases, and no obvious abnormality was seen in imaging examination.

### Double ETG

**Figure 10:**
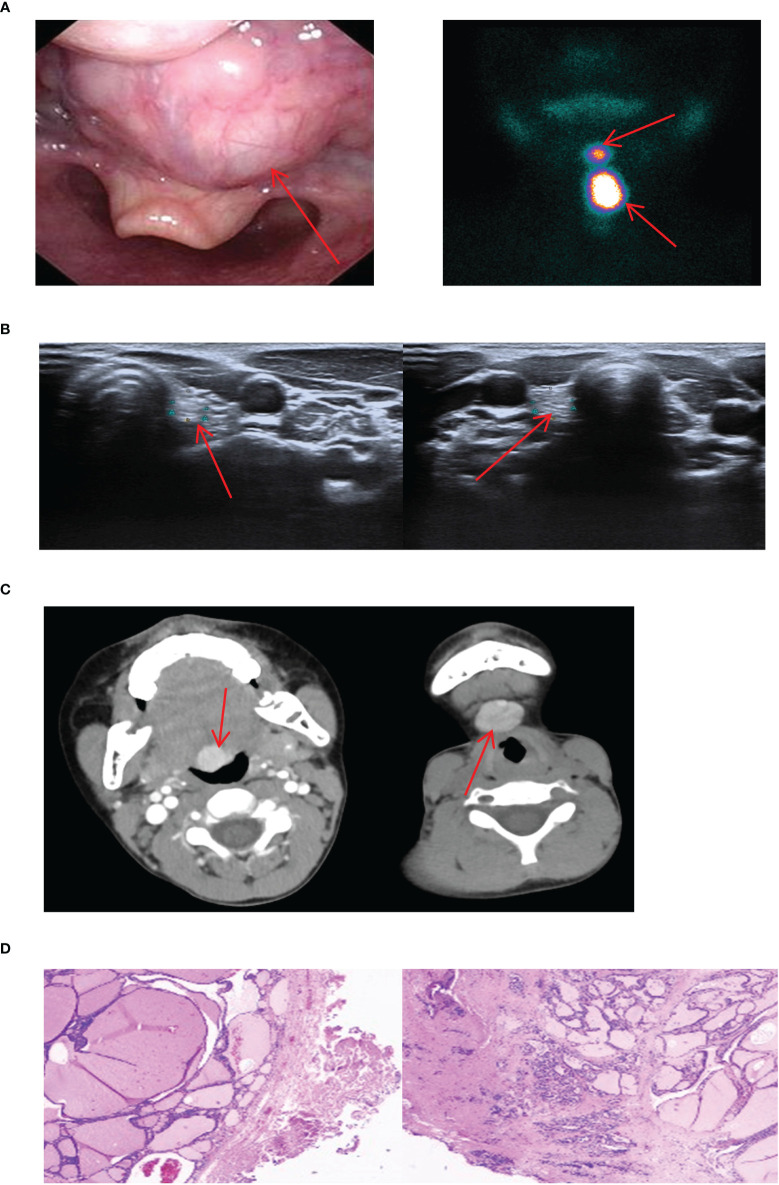
Imaging manifestations and pathological findings of the lingual + submandibular dual ETG. **(A)**: The laryngoscope showed a large, reddish, round new creature at the root of the tongue with a smooth surface and mobility. Static imaging of thyroid gland respectively showed a circular abnormal aggregation in the distribution of radioactivity at the root of the tongue and the submandibular position. **(B)**: The thyroid ultrasound showed the bilateral small thyroid glands. **(C)**: CT respectively showed a circular mass with clear boundaries at the root of the tongue and the submandibular position. **(D)**: The postoperative pathology of the lingual + submandibular dual ETG both showed the nodular goiters.

**Figure 11:**
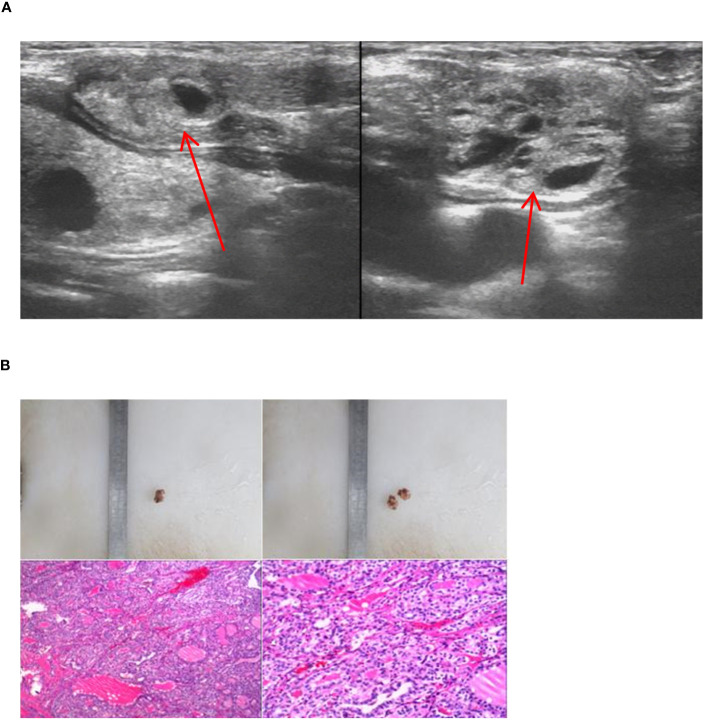
Imaging manifestations and pathological findings of the lateral cervical dual ETG. **(A)**: A cystic-solid mass was seen respectively at the extremities sternalis of the left sternocleidomastoid and the right side of suprasternal fossa, with smooth edges. **(B)**: The postoperative pathology of the lateral cervical dual ETG both showed nodular goiters. **(A, B)** were of the same patient.

In our study, we encountered 5 cases of double ETG, comprising 3 females and 2 males, with ages ranging 5-62 years old. These included 3 cases of double ETG at the base of lingual and submandibular and 2 cases were located in the lateral cervical. In the 3 cases of double ETG at the base of lingual and submandibular, all the patients presented with hypothyroidism. Two of them had noticeable masses protruding from the skin surface during the neonatal period, and they were treated with THRT 5 and 10 years later, respectively (**Figure 10**). The third patient presented with laryngeal foreign body sensation and snoring symptoms 3 years after the discovery of a painless and suspicious mass in the neck. As a result, he underwent surgical resection, followed by THRT. In another two cases, the lateral cervical double ETG was detected as suspicious masses during regular follow-up examinations after previous thyroid surgeries (**Figure 11**). They both had surgeries and were confirmed as nodular thyroid goiters with postoperative pathology. Subsequently, they maintained normal thyroid function by undergoing THRT. The mean follow-up time for the 5 cases of double ETG was 73.4 months. They all had normal thyroid function, and no recurrence was observed during the follow-up time.

## Discussion

ETG, a congenital embryonic developmental disorder, is one of the major causes of thyroid underdevelopment. Worldwide, there have been fewer than 10,000 reported cases, making it a rare condition and often found in death autopsies (11). ETG can develop at any age and is more prevalent in females. In our study, 78.7% were females, consistent with previous studies. This gender predisposition may be attributed to the fact that females require more thyroid hormones during puberty, menstruation and pregnancy (3, 12). In1869, Hickman reported the first case of ETG at the base of the lingual root. In subsequent reports, ETG has been found in the pituitary gland, larynx, trachea, submandibular and lateral neck region (13, 14). The thorax is found in the lungs, breast, heart, thymus, etc (15–18). The digestive system was found in stomach, gallbladder, hepatic hilum, pancreas, etc (19–21). Urinary and reproductive systems are seen in adrenal glands, ovaries, fallopian tubes, uterus, etc (22, 23). ETG of this study are focused on the lingual, cervical, mediastinal and ovarian. Lingual ETG accounted for the highest proportion, with up to 20 cases (42.55%), followed by the cervical ETG. Similar to the previous study, ACTG was more common than ABTG. Furthermore, since the thyroid gland is composed of multiple parts during embryonic life, ETG may occasionally present as the much rarer double or even triple ETG, typically observed in the oral cavity and neck. We report 5 cases of double ETG in the head and neck (24–27). It is worth noting that thyroid surgery is strongly associated with ETG formation. Batsakis suggests that a history of neck surgery and trauma can lead to neck muscle movement to segment the normal thyroid gland. However, further investigation is needed to verify this association in a larger number of cases (3).

The clinical presentation of ETG is generally nonspecific and insidious, and the majority of patients with ETG are asymptomatic. In cases who are clinically evident, symptoms are usually associated with size, location, type of pathology, and the presence or absence of thyroid function abnormalities (1, 6). Among symptomatic patients, foreign body sensation is usually the prevalent complaint, which can cause irritating symptoms like coughing in less severe cases. As the disease progresses, it can leads to localized pressure pain. Although lingual ETG is the most common ETG, it’s noteworthy that cervical ETG (20 years) is detected earlier than the mean age of lingual ETG (30.5 years). Conversely, ovarian ETG tends to be identified at a later age. Symptoms of ETG are variable depending on their location within the body. For example, patients with tracheal ETG may experience dyspnea and hemoptysis. The thoracic ETG may cause dysphagia and superior vena cava syndrome, while abdominopelvic organ ETG may cause bleeding, abdominal pain, and obstructive jaundice (28–32). Although most ETGs are benign and asymptomatic, the associated symptoms and the possibility of cancer should not be ignored. hese cases necessitate prompt management or active monitoring and observation.

When ABTG is the sole source of thyroxine secretion, its secretion is usually inadequate. In contrast, ACTG sometimes results in hypothyroidism due to inadequate blood supply to the normal thyroid gland, which may lead to the appearance of a small and non-functioning thyroid gland *in situ*. This explains the occurrence of hypothyroidism in 1/3 of the patients with ETG in our present study. In addition, 4 cases in this study presented with hyperprolactinemia. After excluding other factors, we believe that the patients’ thyroxine insufficiency may feed back to the pituitary gland, leading to an increase in thyrotropin-releasing hormone. This, in turn, stimulates the secretion of prolactin and causes the development of hyperprolactinemia (33). Imaging and pathology are essential in the diagnosis of ETG. In cases of suspected ETG, preliminary ultrasonography of both the thyroid and ectopic masses is indispensable and should not be omitted. Ultrasonography is cost-effective and safe, and should be the preferred choice for ETG (34). Additionally, ^99^Tc^m^, I^131^ or I^123^ thyroid static imaging is crucial not only for accurately showing the presence or absence of the thyroid gland *in situ* and its function but also for helping localize the ETG. Due to the advantages of good imaging, low cost, low radiation, and safe to use in children, ^99^Tc^m^ is more widely used (7, 20, 35). The gold standard for diagnosis of ETG is pathological examination, and FNAC is the preoperative method with a diagnostic accuracy of over 90%. Results suggesting malignant types such as PTC should be operated on as soon as possible (11). CT and MRI play an important role in detecting ETG that migrate away from the thyroid, occasionally intracranial, thoracic, abdominopelvic, and concealed locations. These imaging modalities can clearly show the size and location of the mass, and whether or not it is infiltrated and adherent (11, 36). In addition, specialized endoscopic procedures such as laryngoscopy and esophagoscopy can visualize ETG. However, due to their invasive nature and high cost, these procedures should only be employed when deemed necessary. It is vital to note that ETG is easily misdiagnosed as lingual root cysts and thyroglossal duct cysts. Therefore, carefully distinguishing these conditions from others is crucial to minimize diagnostic errors (11, 28, 37).

Treatment should comprehensively consider the symptoms, type, its benign or malignant nature of the ETG, as well as the patient’s age and thyroid function. As a matter of attention, ETG can develop the same pathological alterations as normal thyroid tissue, such as goiter, thyroiditis and carcinoma (11). Consequently, ultrasound and thyroid function need to be followed up over time to catch problems and provide timely intervention, regardless of the treatment modality. In cases of benign ETG, treatment should be based on the type of it. Particularly, the ABTG, the only tissue producing thyroxine in the body, wrongly cut could hinder growth and development especially in juvenile patients. Therefore, asymptomatic patients may continue to be under observation, and those with hypothyroidism can be given THRT (2). In case of compressing adjacent organs, I ^131^, thermal ablation, and surgical resection are options (5, 13). For the ACTG, the treatment for asymptomatic and hypothyroid patients aligns with that for ABTG. However, the difference is that ACTG is more prone to grow in size, and TSH should be controlled <2.5mU/L (6). If ETG is malignant, the management should follow the principle of thyroid tumor, with attention to the thyroid *in situ* (31). In our study, the only case of PTC was observed in a lingual ETG. The patient underwent surgical resection, followed by TSH suppression therapy, and no abnormalities of ultrasound and thyroid function were detected at the end of the follow-up.

## Conclusion

In summary, ETG is a rare clinical condition characterized by abnormal thyroid development. It predominantly affects the head and neck region and often manifests without specific symptoms. When clinicians encounter unidentified masses, it is crucial to realize the possibility of ETG and conduct necessary examinations to establish an accurate diagnosis and prevent misdiagn. The primary diagnostic tool for ETG is thyroid ultrasound, which aids in confirming the presence of thyroid tissue. Additionally, thyroid function tests are essential for evaluation. Thyroid Static Imaging plays a pivotal role in localizing and characterizing ETG. FNAC or postoperative pathology result are considered as the gold standard for diagnosis. Once ETG is diagnosed, treatment options differ by clinical symptoms and nature of pathology. Asymptomatic and benign lesions may be suitable for observation or THRT, based on thyroid function status. Surgery is suggested for malignant lesions or when symptoms are severe and significantly impact daily life and appearances. Regular follow-up, including ultrasound and thyroid function tests, is necessary for all ETG patients to monitor disease progression effectively.

## Scope statement

Ectopic thyroid gland is rare in endocrine disorders, and our study retrospectively analyzed the comprehensive course of 47 cases of ETG with the largest single-center sample size in the world to date. Demonstration of specific clinical presentations of ETG in various sites and, most importantly, follow-up of all patients is unparalleled. The treatment method tends to vary considerably, and individualization of the choice of observation, surgery, and drug therapy is particularly critical. Although carcinoma is extremely unusual, benign tissue also tends to be accompanied by hypothyroidism as well, requiring long-term follow-up and timely intervention. Our research will provide valuable insights into the diagnosis and treatment of ETG, with the goal of developing clinically relevant diagnostic and management protocols.

## Data availability statement

The raw data supporting the conclusions of this article will be made available by the authors, without undue reservation.

## Ethics statement

The studies involving humans were approved by the Medical Ethics Committee of Zhengzhou University (Approval No.2021-KY-0202-002). The studies were conducted in accordance with the local legislation and institutional requirements. The human samples used in this study were acquired from primarily isolated as part of your previous study for which ethical approval was obtained. Written informed consent for participation was not required from the participants or the participants’ legal guardians/next of kin in accordance with the national legislation and institutional requirements. Written informed consent was obtained from the individual(s), and minor(s)’ legal guardian/next of kin, for the publication of any potentially identifiable images or data included in this article.

## Author contributions

MG: Writing – original draft. QH: Writing – original draft. LL: Writing – review & editing. FJ: Writing – review & editing. YD: Writing – review & editing. QS: Writing – review & editing. XQ: Writing – review & editing.
